# What We Know—and Need to Know—About Nursing PhD Programs and Influences on the PhD–Faculty Pipeline: A Scoping Review

**DOI:** 10.1111/jan.70506

**Published:** 2026-01-30

**Authors:** Olivia M. Halabicky, Joshua Porat‐Dahlerbruch

**Affiliations:** ^1^ Department of Health Behavior and Clinical Sciences University of Michigan School of Nursing Ann Arbor Michigan USA; ^2^ Department of Acute and Tertiary Care University of Pittsburgh School of Nursing Pittsburgh Pennsylvania USA

**Keywords:** nurse faculty, nurse faculty shortage, PhD program, PhD‐faculty pipeline

## Abstract

**Aims:**

To identify: (1) current evidence and gaps of PhD program components influencing PhD students' career outcomes; and (2) methods and tools used to assess the relationships between PhD program components and career outcomes.

**Design:**

PRISMA scoping review.

**Methods:**

Search terms included PhD nursing students, PhD education, PhD‐prepared nurse, PhD in nursing, nursing faculty, and assistant professor. Studies empirically assessing PhD program components and career outcomes (e.g., desires, attitudes, actual employment) were included. Two researchers conducted screening, data extraction, and inductive content analysis.

**Data Sources:**

PubMed, Scopus, and CINAHL in October 2025, without year and geographic location restrictions.

**Results:**

The search yielded 379 studies. After title, abstract, and full‐text screening, 13 studies were included. Analysis resulted in 10 factors spanning four categories: program preparation, readiness and satisfaction, impressions of the faculty role, and program support.

**Conclusion:**

Experiences in the PhD program likely influence students' desire to pursue academia. While this review synthesized influential factors, given significant gaps in the literature, there are likely more factors influencing student career desires. A more robust understanding of the factors during the PhD program which influence career outcomes is needed.

**Patient or Public Contribution:**

This study did not include patient/public involvement in design, conduct, or reporting.

## Contribution to the Wider Global Clinical Community

- Synthesis of nursing PhD program components influencing student career outcomes.
- Importance of targeting all three stages of the PhD‐Faculty Pipeline for the Nursing Profession.
- Discussion of areas of future research and tools needed to identify associations between PhD program components and career outcomes, which can be used to develop interventions.

## 1 Introduction

There are 2200 full‐time faculty job openings across Schools of Nursing (SoNs) in the United States (American Association of Colleges of Nursing 2022b). The demand for SoN faculty is expected to grow as one‐third of nursing faculty in both undergraduate and graduate programs will retire by 2025 (Fang and Kesten 2017; Fang et al. 2024). As it stands, the PhD‐prepared nursing workforce will not mitigate the faculty shortage—fewer than 1% of US nurses possess a PhD (American Association of Colleges of Nursing 2022a). From 2021 to 2022, there was a 4% decrease in nursing PhD enrollments (American Association of Colleges of Nursing 2022b), and researchers predicted that PhD enrollments and graduations will decline 20% by 2032 (Halabicky et al. 2024). This trend is seen globally, where PhD enrollments in nursing and other fields are also declining (McKenna and Thompson 2025).

Perhaps most importantly, nursing PhD students are increasingly pursuing careers outside of academia (Research‐Focused Doctoral Program Pathways to Excellence Task Force 2022). The National Research Council (2006) reported that only about half of PhD‐prepared nurses enter academic positions after graduation (National Research Council and Institute of Medicine Committee 2006). This statistic, nevertheless, is two decades old; it is thought that fewer nursing PhD graduates are entering academia today (Broome et al. 2023). Given declining numbers of PhD graduates, those pursuing academia, and the growing faculty shortage, strategies are needed to ensure growth in the PhD‐Faculty pipeline.

The PhD‐Faculty pipeline is the trajectory from pre‐PhD student to nursing faculty member and consists of three distinct periods: (1) pre‐PhD program, (2) PhD program, and (3) post‐PhD program transition to faculty roles (Stanfill et al. 2019). Evidence‐based strategies at each stage of the pipeline are necessary to combat the nursing faculty shortage. Several strategies have addressed the first stage of the pipeline aimed at increasing enrollment in PhD programs by providing opportunities for BSN students to engage in research, increasing excitement for pursuing a research career, and developing specialised programs to support earlier entry to PhD programs (e.g., BSN‐to‐PhD pathways) (Ayoola et al. 2021, 2017; Granner and Ayoola 2021; Muñoz 2022). Similarly, much research has focused on downstream factors, at the last stage of the pipeline, which attempt to keep nurses in academia through mentorship models supporting early‐career research faculty, robust onboarding for new faculty, reducing burnout, and promoting a culture of civility within SoNs (Jones et al. 2026; Melnyk et al. 2023; Miller et al. 2024; Park and Kang 2023).

**FIGURE 1:**
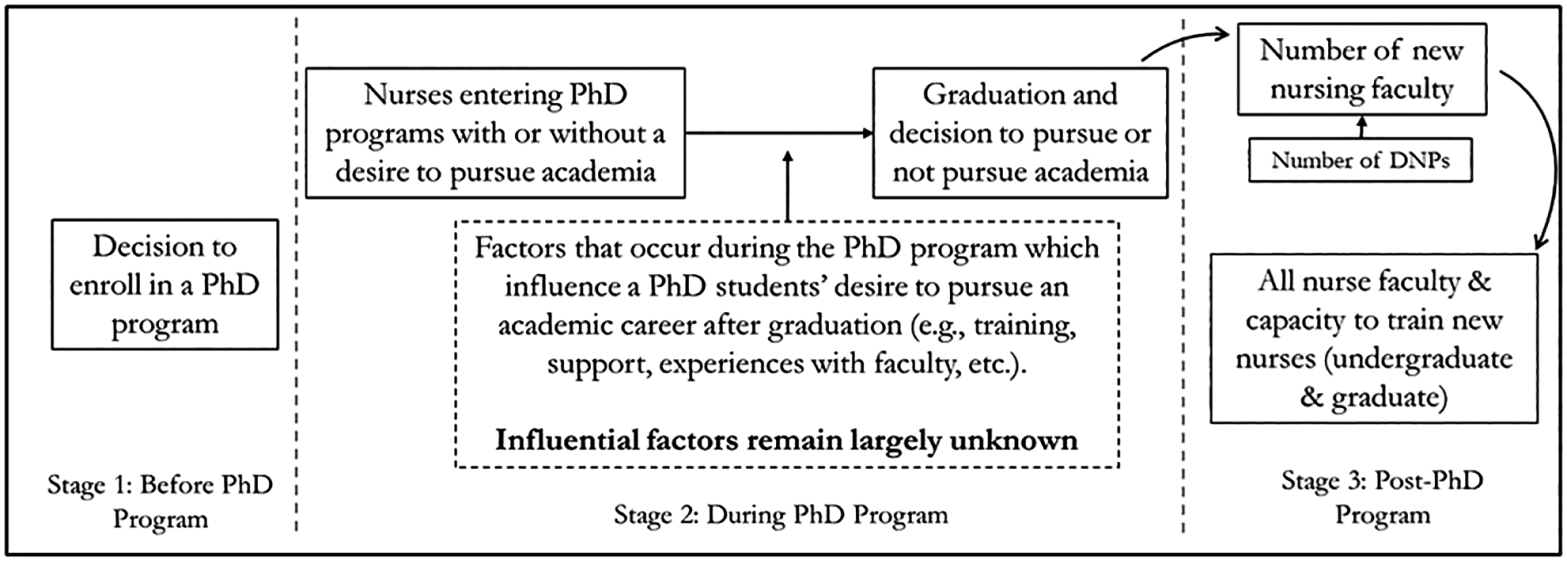
Conceptual model: The PhD‐faculty pipeline and the decision to pursue academia.

Indeed, interventions to combat the nursing faculty shortage are needed at every stage of the pipeline to increase PhD program enrollment (Stage 1), cultivate interest in academic careers (Stage 2), and promote faculty retention (Stage 3). Nonetheless, there is a greater dearth in research from the second stage of the pipeline, a period when PhD students form opinions towards academia and make future career decisions. As a result, there is limited understanding of factors during a PhD program that influence students' desire to pursue careers in academia (Figure 1).

Due to the lack of evidence, those interested in increasing the number of PhD graduates pursuing academia rely on recommendation articles or non‐generalizable reports from specialised programs (Broome et al. 2023; Han et al. 2022; Research‐Focused Doctoral Program Pathways to Excellence Task Force 2022; Stanfill et al. 2019). Some influential factors may include peer and faculty mentorship, supportive research experiences, and meaningful teaching experiences (Fang et al. 2016; Killela et al. 2024; Lorenzetti et al. 2019; Porat‐Dahlerbruch et al. 2021). Still, it is unclear whether these factors resonate with current PhD students and, perhaps more importantly, if strategies addressing these factors result in an increased number of graduates pursuing academia.

### 1.1 The Review

To develop interventions during Stage 2 of the PhD‐Faculty pipeline that support and encourage PhD students to pursue academia, thereby combating the nursing faculty shortage, we must understand the factors within the PhD program influencing PhD students' career goals, attitudes, and later employment. The first step to launching this line of investigation is a scoping review to survey the literature to identify what is already known in this area as well as gaps.

### 1.2 Aim(s)

This scoping review has two aims: (1) to identify current evidence and gaps in understanding of PhD program components which may influence PhD students' career outcomes; and (2) to identify methods and tools, if any, used to assess the relationships between PhD program components and career outcomes.

## 2 Methods/Methodology

### 2.1 Design and Search Methods

We followed recommendations by the PRISMA Extension for Scoping Reviews methodologies (File S1) (Tricco et al. 2018). Searches were performed in PubMed, Scopus, and CINAHL in October 2025. We included search terms related to both nursing PhD students/education and nursing faculty. Search terms were broad to ensure inclusion of the full spectrum of PhD program components. Related search terms included PhD nursing students, PhD education, PhD prepared nurse, PhD in nursing, nursing faculty, and assistant professor. From these, synonym strings were created which included database‐controlled vocabulary terms where available (i.e., MeSH, mainsubject terms). Specific search strings for each database are shown in Table S2.

### 2.2 Inclusion and/or Exclusion Criteria

After the search, studies were screened by title and abstract and then by full text against the inclusion and exclusion criteria by two reviewers using Covidence software (Covidence 2022). To be included in the review, studies had to examine linkages between PhD program components and career outcomes. We defined PhD program components broadly to capture a multitude of factors occurring during this time period. These could include specific curriculum or activities within a program, support offered by a program, student perceptions of program activities, and self‐perceptions of competency and skills gained within a program. Career outcomes were defined broadly and included student career goals, attitudes towards academic careers, and actual employment data. Importantly, studies had to include both a measure of PhD program component *and* career outcomes; studies that reported only on a PhD program component (e.g., curriculum change) and did not include a measure of career outcomes were excluded. Included studies had to be peer reviewed, apply an empirical qualitative, quantitative, or mixed‐method approach, and be reported in English. Studies could have included prospective data (i.e., in this context, a student reflecting on current program experiences and future career goals) or retrospective data (i.e., in this context, current faculty reflecting back on PhD program experiences). There were no limitations on years nor geographical location. Editorials, opinions, or commentaries without empirical study were excluded.

### 2.3 Data Abstraction

Extracted data included sample characteristics, years of data collection, sample size, stated purpose of the study, qualitative or quantitative assessments, measurement tool(s) used and data source, analysis methods, PhD program measure, career outcome measure, and relevant findings. One reviewer abstracted the above data, which was verified by the second reviewer using a Covidence extraction tool (Covidence 2022).

### 2.4 Data Analysis

Data analysis initially included descriptive data of the included studies, such as years of data collection, location, data source, study methodology, etc. These characteristics were summarised by frequency counts in associated tables. The measures of PhD program components and career outcomes were defined and organised by similarities between studies. Summaries of these measures were presented numerically in a table.

Given the wide breadth of study designs, an inductive content analysis method was used to analyse the included studies. This process included reading the included studies, organising, and forming categories based on the similarities and differences in the study data (Kyngäs 2020). One reviewer became fully immersed in the data by reading and organising the included studies and extracting characteristics of each study to form themes. The two reviewers met to identify common themes throughout the studies and develop categories and subcategories of factors influencing student career outcomes. Data were summarised numerically in table form for each identified category and subcategory, as well as narratively. Both reviewers continuously reviewed the synthesised themes, which informed the identified categories and subcategories. Given the wide scope and types of data, we did not include a critical appraisal of the included studies, as is acceptable in scoping reviews (Peters et al. 2015).

## 3 Results/Findings

### 3.1 Search and Selection Results

**FIGURE 2:**
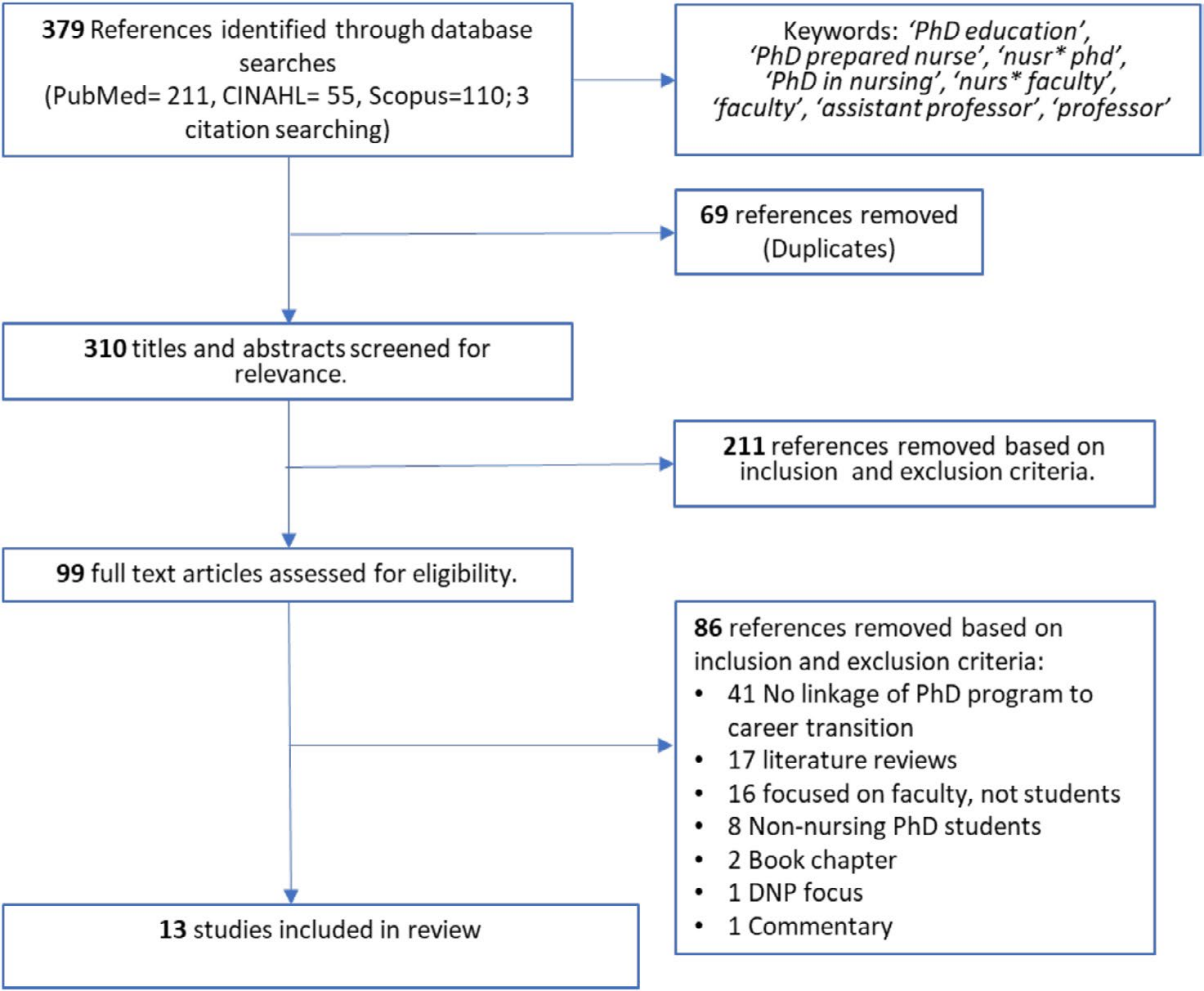
PRISMA diagram.

A total of 379 sources were identified initially—69 duplicates were removed, and 310 remained for screening. References were uploaded to Covidence for screening by two independent reviewers (Figure 2) (Covidence 2022). During title and abstract screening, 211 sources were removed based on inclusion and exclusion criteria. After full‐text screening, 86 sources were excluded, leaving 13 studies. The main reason for exclusion was not linking PhD program components to career outcomes (i.e., goals, attitudes, or employment) (*N* = 41). Other reasons included a focus on faculty instead of student experiences (*N* = 16), being a literature review (*N* = 17), including samples of non‐nursing PhD students (*N* = 8) or DNP students (*N* = 1), and being non‐peer reviewed such as a book chapter (*N* = 2) or commentary (*N* = 1).

### 3.2 Study Characteristics

Overall study characteristics (e.g., location, years of data collection, purpose, methodology, and findings) are included in Table 1. General characteristics for each study are tabulated in Table 2. While not an exclusion criterion of the review, all included studies were based in the United States, with a majority including participants from multiple states (*n* = 9), and one study each from Maryland, North Carolina, Wisconsin, and Alabama. There were no restrictions on year, and most studies collected data from participants between 2011 and 2020 (*n* = 10), with a few from 2000 to 2010 (*n* = 3), and more recently from 2021 to present (*n* = 2).

**TABLE 1:** Summary of included studies.

Author	Sample	Purpose	Design	Tool used	PhD program component	Career outcome	Findings
Daw et al. (2021)	Maryland, Nurse Support Program Jo Cohen Graduate Nurse Faculty Scholarship (GNF), *N* = 250 (Jan–Oct 2019 data collected for years 2016–2019)	To analyse the effectiveness of a state‐wide faculty financial support program in supporting and creating nurse educators	Quantitative	None	Financial support for all tuition and mandatory fees for completing graduate education in Maryland. Degrees include MSN/MS, PhD, DNP, and EdD programs which prepare nurses to become nurse faculty. Recipients are required to work for 1 year as a full‐time nurse faculty member, hospital educator, or in an approved educational role in Maryland (2 years if part‐time)	Graduation and fulfilment of work obligation in a nurse faculty role	Of the 250 GNF awardees, 65 have completed their graduate degree, while the remaining 186 were expected to confer within 1–5 years from 2019. Only three (1.6%) of these students were pursuing a PhD. Of those graduated, 30% work full time (70% part time) at a university (*N* = 94), community college (*N* = 80), hospital educator (*N* = 49), or long‐term care/outpatient (*N* = 27). If awardees do not complete their service obligation, the award is converted to a loan. Since 2016, 10% of awardees have repaid their awards ($1,105,755)
Dreifuerst et al. (2016)	Multistate (PA, SC, ND, IL, OR, AZ, ID), US: Current and recent graduates of doctoral programs including PhD and DNP, *N* = 398 students (196 PhD, 202 DNP) and *N* = 150 recent graduates (75 PhD, 75 DNP) (2016, graduates since 2010)	To report findings from a multisite study exploring factors which influenced nurses in completing their doctoral programs and pursuing/retaining nurse faculty roles	Mixed‐methods	92‐item, branching by selection questionnaire. Included 4‐point Likert scale responses and open ended responses	Likert responses for individual questions were analysed in categories (1) time, (2) money, (3) program confusion, (4) faculty role. Content analytic summary of opened ended responses was used to describe themes within these categories Questions within the ‘faculty role’ category included (1) “How well do you feel your doctoral education is preparing/prepared you in assuming a nursing faculty role?” (2) How many courses related to the faculty role do you plan/did you take in your doctoral program”	Questions within the ‘faculty role’ category included (1) indicating current position (full time or part time faculty) (2) Did you begin your doctoral program with the intent of becoming a doctorally prepared nursing educator?” Open ended responses were also included related to career desires/intentions	52.13% of current PhD students and 25% of PhD graduates indicated being full‐time faculty. 79.38% of current PhD students and 86.30% of PhD graduates indicated beginning their doctoral program with the intent of becoming a nurse educator. 86.91% of current PhD students and 82.19% of PhD graduates indicated that their program is/did prepare them for a nurse faculty role. 41.61% of students and 24.98% of graduates reported taking one but no more than two courses related to the faculty role with 22.94% of students and 25.62% of graduates reporting these courses related to curriculum, evaluation, or teaching practicum. Considering the open‐ended responses, almost half of respondents indicated dissatisfaction with their program in their preparation for a faculty role
Ellenbecker et al. (2017)	Multistate, US: 22 schools with accredited PhD programs as listed by the AACN, *N* = 204 survey respondents (Fall 2012, graduates since post‐2002)	To describe outcomes of nursing PhD programs and examine relationships between the characteristics of individual nursing students, the PhD programs they attend, and program outcomes	Quantitative	41‐item survey with information on program activities, online vs. in person program, resources, and post‐graduation research activities (presentations, grants submitted and awarded, publications, etc.)	Working as an RA, program format, working as an RN during program, presenting or publishing as a student, and age at graduation	Self‐reported involvement in “research career”	Working as an RA, working less as an RN while a student, and giving presentations and publishing as a student were positively associated with having a research career. In adjusted regression models, fewer hours worked as a nurse (*p* = < 0.001), student presentations (*p* = 0.019), and postdoctoral experience (*p* = 0.038) were significantly associated with a self‐reported research career
Fang et al. (2016)	Multistate, US: *N* = 933 PhD students, Nationally representative sample, collected in 2013	To report on doctoral students' program experiences and career decisions	Quantitative	Online survey (investigator developed). Mix of multiple choice, Likert, and open‐ended questions	Factors related to participation research, teaching, and leadership activities. Mentorship and financial support were also assessed. Respondents were asked to indicate whether a particular experience increased, decreased, or did not impact their interest in academic nursing	Desire to pursue future career in academia, non‐academia, or undecided	Of the 933 respondents, 51.4% were employed by a teaching/research institution at the time of the survey. A total of 676 (72.5%) reported desiring a career in academia, while 101 did not desire an academic career and 156 were undecided. Resulting analyses divided the sample into these three groups and assessed whether PhD program experiences and perceptions differed between the groups and influenced group self‐selection Among many results, 76.3% of respondents who desired an academic nurse career reported that interactions with faculty during their doctoral education influenced their desire for an academic career, with those who desired an academic career reporting greater confidence. Conversely, of those who desired a non‐academic career, 31.7% reported interactions with faculty during their education dissuaded them from an academic nursing career The level of confidence in many faculty like activities significantly varied between those who desired and those who did not desire academic careers including developing curricula, teaching nursing courses, and teaching clinical courses. The levels of confidence for conducting independent research, publishing findings, and reviewing and writing grant proposals were not different between groups A perceived increased impact of teaching, research, and leadership experiences on career plans for academic nursing were associated with a 249.9% increase in the odds of planning for an academic career. Increases in the impact of teaching, perceived contribution of nursing research to patient care, and perceived financial compensation in academic nursing on plans for an academic career all increased the likelihood of having an academic career
Giordano et al. (2023)	Multi‐institution, US: Robert Wood Johnson Foundation Future of Nursing Scholars program, *N* = 179 scholars who graduated between 2017 and 2022 and *N* = 60 faculty members (September 2020–March 2021)	To assess program experiences and career outcomes via CV review of scholars completing the Future of Nursing Scholars program and through interviews with faculty at participating institutions	Mixed‐methods	None	School participation in the Future of Nursing Scholars program. Assessed through 60 min interviews with faculty representatives from participating institutions	Employment settings and postgraduate publications, presentations, journal reviews, and awarded grants. Data collected through CV review of previous scholars	Prior to enrollment, most scholars were enrolled in healthcare settings. After graduation, a majority of graduates were employed by a university or college wither as a postdoc or in an academic role, evidencing a significant employment role transition (×2 = 34.24, *p* < 0.001). Engagement in research activities also significantly increased post‐graduation. Considering faculty responses, most participating schools (60%) underwent curriculum changes to accommodate the 3‐year requirement of the program, with 27% moving to 12 month curriculum to accommodate the timeline. Faculty were mixed on the 3‐paper vs. traditional format dissertation, which may actually take longer to complete
Lee et al. (2022)	Multi‐institution, US: Full‐time nurse faculty with a PhD or DNP in nursing with ≤ 4 years of teaching experience after doctoral program graduation, *N* = 149, *N* = 71 held a PhD (March 2015)	To examine factors and predictors which are associated with new nurse faculty's intent to leave academia.	Quantitative	Transition to the Nurse Faculty Role Survey (TNFRS) including four domains (1) doctoral teaching preparation, (2) nurse faculty institutional support, (3) faculty job satisfaction, (4) intent to leave current nursing academic position	Doctoral teaching preparation: six questions related to coursework on teaching, credit hours taken, and teaching experience including mentored internships, teaching or research assistantships, and graduate teaching appointments.	Intent to leave nursing academic position: Question indicating how likely participants were to leave their academic position. If participants responded affirmatively, they were additionally asked the timeframe for their departure (1–2 years, 3–4 years, 5–6 years, or more) and their reasons for leaving	46% and 8% of participants were tenure track or tenured, respectively. 27.52% reported they were likely to leave their current academic position, 68.2% within the next 6 years and 26.4% within the next 1–2 years. In adjusted logistic regression models, doctoral experiences related to coursework and teaching were not significantly associated with intention (odds) to stay in their academic role
Lewallen and Kohlenberg (2011)	North Carolina, US: University of North Carolina at Greensboro School of Nursing doctoral program students who completed a 6 credit course on the faculty role (Pre–2011)	To describe and evaluate the impact of a Faculty Role course implemented within nursing PhD curriculum	Narrative	None	Two courses aimed at preparing for the faculty role. Course I is didactic including information on tenure, academic outcomes, regional and specialised accreditation, academic leadership, and evolution of nursing education. Roles of nurse scientists in industry also included. Other topics include mentorship, peer review of manuscripts, identifying grant funding, strategic career planning, and marketing oneself for a desired position. Two main assignments are completed including writing a solo‐authored manuscript and generating a career portfolio including a CV, teaching philosophy statement, 5‐year career plan with goals and timeline, potential projects to submit for funding, and possible grant‐funding agencies and proposal guidelines. Course II is a 90‐contact hour internship in either academia or industry. Internship activities could include teaching, revising and submitting an NIH grant, co‐authoring manuscripts, writing an individual grant, and learning about institutional accreditation	Student self‐reported experience with the course and feelings towards preparation for nurse faculty role	The authors reported “To date, approximately half the students have acquired positions in academia or industry because of [the] internship experience”. At the time of the study, approximately 14 students had completed the program within a 3–4 year timeframe. Students report “the course helped me identify my purpose/role as a nurse in academia”. Full survey results are not reported
McNelis et al. (2019)	Multistate, US: *N* = 24; 12 current students (6 DNP & 6 PhD) as well as 12 recent graduates within the previous 2 years (6 DNP & 6 PhD) (2016, graduates since 2010) Secondary analysis of Dreifuerst et al. (2016)	To understand nursing doctoral education experiences related to teaching preparation and career expectations	Qualitative	Individual semi‐structured interviews. Secondary analysis of data previously collected from larger mixed‐methods study on doctoral nursing education	Themes included (1) met and unmet expectations of programs and (2) equivocal preparation for teaching	Expectation to hold a “research career”	A majority of PhD respondents reported they did not receive formal or standardised instruction related to pedagogy. They, however, anticipated they would receive this and many expressed concerns that this lack of preparation would impact their success in academia. Overall, there was a reported disconnect between the expectations of doctoral education and actual skills required for a faculty role
Nehls et al. (2016)	Wisconsin, US: Students admitted to the PhD program at the University of Wisconsin‐Madison over a 10‐year period, *N* = 84, includes three groups (1) immediate entry post‐BSN, (2) 1 year clinical experience, (3) delayed entry with masters degree and 1+ years work experience (Fall 2002–Fall 2011)	To compare outcomes of students who were early entry to PhD programs and those who began their PhD later in their education/career	Mixed‐methods	None.	Review of program database: comparing three groups of students (1) immediate entry post‐BSN, (2) 1 year clinical experience, (3) delayed entry with masters degree and 1+ years work experience	During an interview, participants were asked “What are your thoughts about becoming a faculty person?”. Postgraduation employment was assessed using school database information	In qualitative responses, faculty mentorship was reported as key to the progression as a nurse researcher. Students from all three enrollment groups expressed concern about entering a faculty position, and especially a lack of teaching preparation. “I have people recruiting me right now for academic positions. I'm not even sure what that means. Or what do I do?”. Being a teaching assistant or guest lecturing was not reported as being sufficient to prepare graduates for a faculty role. Early‐entry (BSN) students expressed concerns about lack of clinical experience. Regardless of entry timing, students who sought out non research‐intensive environments reported that the academic environment did not look appealing. Regardless of PhD entry time, students had similar levels of research productivity and 84% of graduates held faculty or postdoctoral positions. However, a higher percentage of early‐entry graduates were in postdoctoral fellowships or faculty positions in research‐intensive universities (57% early entry, 44% mid‐entry, 31% delayed entry)
Nersesian et al. (2019)	Multi‐institution, US: PhD students *N* = 372 (Jan‐July 2016)	To assess characteristics and practices of nursing PhD students, and mentoring characteristics of advisors, which contribute to self‐reported career readiness	Quantitative	Electronic survey tool (investigator developed)	Self‐rated proficiency (research knowledge, scholarly writing, professional presenting, teaching, ethics knowledge, and professional skills), doctoral student characteristics (motivation, organisation, dependability, and openness to suggestions or criticism), advisor characteristics and mentoring practices, characteristics of the school	Self‐reported career readiness on a scale of 0–100	Student reported career readiness levels were used to divide participant responses into three groups: minimally (*N* = 124), moderately (*N* = 134), and highly (*N* = 114) career ready. About half (50.7%) reported a desire for a career post‐graduation that combined research and teaching. Those who rated themselves moderately and highly career‐ready, compared to minimally career ready, had a greater likelihood of having higher self‐rated proficiency, more hours worked per week, more responsibilities outside the PhD program, a co‐advisor, access to both advising and mentoring, and attending a private school. Students who rated themselves highly career‐ready, compared to minimally and moderately, had a greater likelihood of having higher‐rated self‐proficiency, more hours worked per week, more responsibilities outside of the PhD program, a co‐advisor, and attending a private school
Vance et al. (2020)	Alabama, US: University of Alabama at Birmingham School of Nursing PhD students, *N* = 110; *N* = 67 in pre‐alignment group and *N* = 53 in post‐alignment group (2010–2015)	To describe PhD curriculum changes to respond to the AACN 2010 guidance on creating research‐focused doctorate in nursing frameworks and evaluate preliminary student outcomes comparing the pre‐ and post‐ alignment groups	Quantitative	None.	BSN to PhD entry, decreasing time to graduation, curriculum revisions to support timelines and transition to research careers, 3‐paper dissertation option, 6‐credit hours in research immersion coursework, 20 h/week work on mentor research throughout program, revisiting qualifications of PhD faculty members teaching PhD coursework, formal mentorship training for faculty mentors	School database information on student employment: being in a faculty position post‐graduation	At the time of the study, only 33/67 and 13/53 of the pre and post‐alignment groups, respectively, had graduated. There was no significant difference between the pre‐ and post‐alignment groups in the number of graduates in nurse faculty positions. Significantly more post‐alignment graduates received post‐doctoral training. The authors emphasise that the differences in several outcomes, including employment, would become clearer as more students graduated
Vardaman et al. (2024)	Multistate, US: Nurse educators, *N* = 45, decreased to *N* = 25 by June 2023 (April–June 2023)	To provide a perspective of nursing shortage from the view of nursing educators and provide recommendations	Qualitative	None Nominal group technique using highly structured method of gaining data in focus group and research discussions across several meetings. Participants reviewed literature, provided anecdotal evidence, and reaching consensus on themes	“Confidence in Educators” theme from focus groups	Barriers to targeting the nursing faculty shortage theme	Improving faculty confidence was a major theme. “Participants agreed that many nursing faculty express concerns about having had inadequate formal preparation in pedagogy, curriculum development, assessment, and evaluation. Participants felt there is a need for educational institutions to offer a pathway for nurse educators without formal education in pedagogy to obtain a post‐master's or post‐doctoral certificate in nursing education, better equipping them with the skills to successfully fulfil their academic role” (pg. 205)
Xu et al. (2018)	Multistate, US: Direct entry BSN to PhD students or recent graduates, *N* = 4; 2 early career faculty, 1 postdoctoral fellow, 1 current student	To explore characteristics of direct entry BSN to PhD entry student experiences	Qualitative	Participant journal entries on lived experiences, interactively summarise and classify experiences into summative themes using Delphi method	Themes included (1) commitment to science, (2) nursing identity, balancing family and (3) school expectations	Exploring future job prospects	Participants report being very aware that attaining an academic position at a research intensive institution was the expectation. They reported a “lack of fallback options to work clinically” which increased internal pressure to be highly productive during their programs. Despite an understanding that there would be job opportunities, participants expressed concern over their perceived competitiveness for academic positions. Considering their nursing identity “This decision to forego clinical experience and continue with education was not always recognised by other nurses, academics, family, or friends as a wise or commendable career decision. Panellists noted experiences of having their credibility and motivations as a nurse questioned by others within the nursing profession because of their lack of clinical experience” (pg. 32). However, “recognition and affirmation of their decision to pursue a BSN/BS‐PhD degree from supportive mentors and colleagues helped the panellists build a supportive community and better cope with the negative reactions they received from others” (pg. 32)

**TABLE 2:** General characteristics.

Study characteristics	Count (*n*)	Percentage %
Data collection year		
2000–2010	3	23.1%
2011–2020	10	76.9%
2021–present	2	15.4%
Study location		
Multistate	9	69.2%
Maryland	1	7.7%
North Carolina	1	7.7%
Wisconsin	1	7.7%
Alabama	1	7.7%
Study design		
Quantitative	6	46.2%
Qualitative	4	30.8%
Mixed‐methods	3	23.1%
Data type		
Prospectively collected	4	30.8%
Retrospectively collected	6	46.2%
Prospective and retrospective	3	23.1%
Data source		
Survey	6	46.2%
School data	2	15.4%
In‐person interview	4	30.8%
Phone or online interview	1	7.7%
Journaling	1	7.7%
Analysis		
Narrative	7	53.8%
Descriptive	5	38.5%
Modelled associations	4	30.8%

Included studies used a variety of methodologies, with some using only qualitative methods (*n* = 4), some mixed methods (*n* = 3), and the majority quantitative methods (*n* = 6). Most studies used retrospective data and asked participants to think back to their PhD program experiences (*n* = 6). Some studies included current PhD student participants and collected prospective data asking participants about their future career desires (*n* = 4), while few studies (*n* = 3) included both current students and recent graduates, collecting prospective and retrospective data. Among the data collected in these studies, a majority used investigator‐designed survey tools (*n* = 6), many of which were unvalidated. Others utilized school data on graduate employment (*n* = 2), in‐person interviews (*n* = 4), phone interviews (*n* = 1), or journaling responses (*n* = 1). Considering data analysis, many studies included narrative synthesis (*n* = 7), some provided descriptive results from quantitative findings (*n* = 5), and fewer tested associations between a PhD program component and career outcome (*n* = 4).

Multiple PhD program components were identified throughout the studies, including financial support, curriculum changes, participant perceptions, and actual program activities (Table 3). Broadly, studies included tuition support (*n* = 4), large curriculum changes such as BSN‐to‐PhD programs (*n* = 2), specific faculty role preparation courses (*n* = 2), or large programmatic initiatives such as the Robert Wood Johnson Foundation (RWJF) Future of Nursing Scholars program (*n* = 2). Others included participant perceptions of different aspects of their program including perceived teaching preparation (*n* = 2), overall confidence in their preparation (*n* = 2), student perceived competitiveness due to program preparation (*n* = 1), student self‐assessment of their individual characteristics related to productivity (*n* = 1), and alignment of their nursing identity within the program (*n* = 1). Finally, many studies assessed specific activities experienced during a program such as research (*n* = 4), teaching (*n* = 4), leadership training (*n* = 2), mentorship experiences (*n* = 2), and working clinically while completing the PhD program (*n* = 1).

**TABLE 3:** PhD program components and career outcome focus.

PhD program components examined	Count	Source
Financial Support		
Tuition & stipend	4	Daw et al. (2021); Dreifuerst et al. (2016); Fang et al. (2016); Giordano et al. (2023)
Curriculum changes		
BSN to PhD	2	Nehls et al. (2016); Vance et al. (2020)
Preparation for faculty role course(s)	2	Dreifuerst et al. (2016); Lewallen and Kohlenberg (2011)
Large program changes	2	Giordano et al. (2023); Vance et al. (2020)
Perceptions		
Perceived teaching preparation	2	McNelis et al. (2019); Vardaman et al. (2024)
Confidence in preparation	2	Fang et al. (2016); Vardaman et al. (2024)
Perceived competitiveness due to program preparation	1	Xu et al. (2018)
Student self‐assessed characteristics	1	Nersesian et al. (2019)
Nursing identity	1	Xu et al. (2018)
Actual program activities		
Research training	4	Ellenbecker et al. (2017); Fang et al. (2016); Nersesian et al. (2019); Vance et al. (2020)
Teaching training	4	Dreifuerst et al. (2016); Fang et al. (2016); Lee et al. (2022); Nersesian et al. (2019)
Leadership training	2	Fang et al. (2016); Nersesian et al. (2019)
Mentorship	2	Fang et al. (2016); Nersesian et al. (2019)
Working clinically (pre or during)	1	Ellenbecker et al. (2017)

Career outcomes examined	Count	Source
Employment		
Current career	7	Daw et al. (2021); Dreifuerst et al. (2016); Ellenbecker et al. (2017); Giordano et al. (2023); Lewallen and Kohlenberg (2011); Nehls et al. (2016); Vance et al. (2020)
Intent to leave faculty role	1	Lee et al. (2022)
Confidence in current faculty role	1	Vardaman et al. (2024)
Career Intentions and Expectations		
Readiness for faculty career	2	Lewallen and Kohlenberg (2011); Nersesian et al. (2019)
Desire to pursue academia	3	Dreifuerst et al. (2016); Fang et al. (2016); Nehls et al. (2016)
Job prospects	1	Xu et al. (2018)
Expectation to have a research career	1	McNelis et al. (2019)

Career outcomes measured in the studies fell into two broad areas, (1) actual employment, and (2) career intentions or expectations (Table 3). Considering employment, most studies collected data on the current position held (*n* = 7), one assessed participants' intent to leave their faculty role, and another assessed participant confidence in their current faculty role. Within career intentions and expectations, studies inquired about participants' desire to pursue academia (*n* = 3), whether participants felt ready for an academic career (*n* = 2), perceived job prospects (*n* = 1), and perceived expectation to hold a “research career” (*n* = 1).

## 4 Narrative Synthesis of Categories

After conducting a narrative synthesis of the included studies, four categories were identified based on similar patterns and themes (Table 4). These categories, and associated subcategories, represent factors occurring during a PhD program which may be influential in students' desire to pursue an academic career. The categories included: (1) program preparation, (2) readiness and satisfaction, (3) impressions of the faculty role, and (4) program support.

**TABLE 4:** Included study categories and subcategories.

Category/subcategory	Count	Studies
Program preparation		
Coursework on faculty role (research, teaching, leadership)	2	Dreifuerst et al. (2016); Lewallen and Kohlenberg (2011)
Research experiences	4	Ellenbecker et al. (2017); Fang et al. (2016); Giordano et al. (2023); Vance et al. (2020)
Teaching preparation	5	Fang et al. (2016); Lee et al. (2022); McNelis et al. (2019); Nehls et al. (2016); Vardaman et al. (2024)
Programmatic changes	4	Giordano et al. (2023); Nehls et al. (2016); Vance et al. (2020); Xu et al. (2018)
Readiness and satisfaction		
Confidence in skills and readiness	6	Fang et al. (2016); McNelis et al. (2019); Nehls et al. (2016); Nersesian et al. (2019); Vardaman et al. (2024); Xu et al. (2018)
Satisfaction with program	1	Dreifuerst et al. (2016)
Impressions of faculty role		
Attitudes towards academia	2	Fang et al. (2016); Nehls et al. (2016)
Nursing identity/purpose within academia	2	Fang et al. (2016); Xu et al. (2018)
Program support		
Financial protection	4	Daw et al. (2021); Dreifuerst et al. (2016); Ellenbecker et al. (2017); Giordano et al. (2023)
Multifaceted mentorship	6	Fang et al. (2016); Giordano et al. (2023); Nehls et al. (2016); Nersesian et al. (2019); Vance et al. (2020); Xu et al. (2018)

### 4.1 Program Preparation

Program preparation refers to activities or experiences students have within the PhD program that influence their preparation for an academic career. Identified subcategories included completing coursework on the role of a faculty member, research experiences, teaching preparation, and overall programmatic changes.

#### 4.1.1 Coursework on Faculty Role

Two studies reported on specific courses developed to instruct PhD students on the role of academic faculty. Courses varied, but common themes included curriculum on academic leadership, peer review, grant funding, career planning, tenure processes, and mentorship, though studies generally lacked specificity in defining specific curriculum. Current students reported that such coursework helped them identify their own purpose as a nurse within academia (Lewallen and Kohlenberg 2011). Whether or not these students later pursued an academic position is not reported. In another study, students and recent graduates reported whether they took a course related to the faculty role, with 41.6% of current students and 25% of recent graduates reporting taking one or two courses with a tripartite focus on research, teaching, and leadership (Dreifuerst et al. 2016). The specific details of the course content were not collected in this survey, nor was the association between faculty role coursework and current employment in academia tested.

#### 4.1.2 Research Experiences

Involvement in research experiences was a common theme throughout the included studies, though some lacked specificity (*n* = 4). In one, per PhD graduate responses, working as a research assistant (RA), giving presentations, and publishing as a PhD student were all positively associated with having a “research career” (Ellenbecker et al. 2017). Other research experiences included a 6‐credit hour research immersion course and participating in 20 h/week of work on a mentor's research (Vance et al. 2020). This study, when comparing graduates pre‐ and post‐implementation of research activities, found no significant differences in the number of graduates in faculty positions. As not all post‐implementation students graduated at the time of reporting, it is still unclear what impact these research activities had on students' desire to pursue academia. In another study, participation in “research development activities” did not vary between respondents who desired and did not desire academic careers (Fang et al. 2016), though the exact research activities respondents participated in were not reported. A final study reported employment outcomes for participants of the RWJF Future of Nursing Scholars program, which reported a significant shift from clinical work pre‐program to employment at a college or university (e.g., postdoc or faculty) post‐graduation (Giordano et al. 2023). Still, while the RWJF program included specific cohort research training, individual research experiences at the school level varied, which potentially influenced later careers.

#### 4.1.3 Teaching Preparation

Multiple studies touched on the theme of teaching preparation, with many participants reporting an overall lack of experience and preparation (*n* = 5). In a nationwide survey, respondents who identified as desiring an academic career reported having more teaching‐related development activities than those who did not desire an academic career, a difference which may have been self‐selecting (Fang et al. 2016). With “teaching development activities” interpretation left up to the respondent, it is unclear which activities each student experienced and how that influenced career desires. Others felt that their overall lack of teaching preparation would influence their success in academia (McNelis et al. 2019). Some suggested a pathway graduate certificate in nursing education could better equip them to succeed (Vardaman et al. 2024), which may address others' concerns that being a teaching assistant or guest lecturing was not sufficient to prepare for an academic career (Nehls et al. 2016). Still, in a sample of early career faculty, experiences related to coursework and teaching during their PhD program were not associated with intent to leave their current academic position (Lee et al. 2022). It is unclear what specific teaching experiences would prepare future graduates to succeed in academia, though it is clear that more preparation is desired and needed.

#### 4.1.4 Programmatic Changes

Individual programmatic changes included BSN‐to‐PhD programs, implementing 3‐paper dissertations, and 3‐year PhD programs (*n* = 4 studies). When comparing students from (1) BSN‐entry, (2) BSN with 1 year clinical experience, and (3) post‐masters entry to the PhD program, there were no differences in research productivity between groups (Nehls et al. 2016). However, a higher percentage of BSN‐entry graduates were in postdoctoral positions and faculty roles at research intensive universities compared to the other groups, which may suggest supporting earlier entry to the PhD program as a valuable way to capture nurses earlier with high interest in academic nursing. In another program initiative that included BSN‐entry, 3‐paper dissertations, and a research immersion course, significantly more BSN‐entry students were enrolled in postdoctoral positions compared to students enrolled in pre‐implementation of program changes. These results suggest that BSN‐entry students may be more likely to transition to academic careers which emphasize research (Vance et al. 2020), especially considering participants in another BSN‐entry program reported feeling aware that a future academic career was an expectation of their program (Xu et al. 2018). In fact, these findings may reflect BSN‐entry students enrolled due to their desire for a career in academia, given many of these students may forego clinical experience entirely. Other programs, such as the RWJF Future of Nursing Scholars program, integrated large programmatic initiatives (e.g., BSN‐entry, 3‐year PhD, research and leadership training) and have suggested a shift of graduate employment from clinical pre‐program to academic nursing post‐program (Giordano et al. 2023). Still, the assessment of career outcomes for scholars within the Future of Nursing Scholars program is reflective of widescale programmatic initiatives that remained consistent in some areas (e.g., mentorship provided by RWJF Future of Nursing Scholars' staff) and was likely inconsistent depending on school/program (e.g., curriculum, faculty mentorship). It is therefore difficult to disentangle which pieces of the initiative helped best prepare PhD students to enter academia. It is also likely that scholars selected for this program were already more likely to desire a career in academia, which does not detract from the merit of the program itself though may suggest that the results from the program were tied to the selection of students. Additional follow‐up research should be conducted on BSN‐entry programs in which the outcomes of programmatic components are assessed in isolation and combined.

### 4.2 Readiness and Satisfaction

Students' self‐perceived readiness may influence their desire to pursue an academic career. Feelings of ‘readiness’ may stem from both a confidence in skills as well as overall program satisfaction.

#### 4.2.1 Confidence in Skills and Readiness

Confidence in research independence and self‐assessed skills were regularly reported throughout the studies (*n* = 6). Some participants were concerned with their competitiveness on the job market, despite acknowledging the growing faculty shortage (Xu et al. 2018). Others reported a disconnect between their skills development during their program and the actual skills needed to succeed in academia (McNelis et al. 2019), in particular around teaching (Nehls et al. 2016; Vardaman et al. 2024). Self‐rated confidence also had positive impacts. In one study, those who rated themselves as highly career ready had a higher likelihood of self‐rated proficiency in areas such as research knowledge, writing, and professional skills (Nersesian et al. 2019). Whether this impacted their desire to pursue an academic career was not assessed. The level of confidence in faculty‐like activities such as developing curricula and teaching nursing and clinical courses differed between those who identified as desiring or not desiring academic careers (Fang et al. 2016). However, confidence in independent research activities, such as publishing and writing grant proposals, did not differ between these groups, suggesting adequate teaching preparation is key to perceived future success in nursing academia. A lack of clinical experience as it related to confidence in an academic career was also discussed, in particular for BSN‐entry programs (Nehls et al. 2016). Some students reported being questioned by other nurses about their lack of clinical experience, though support from mentors counteracted those negative reactions (Xu et al. 2018). It is still unclear if perceived confidence impacts PhD graduates' decision to pursue academia.

#### 4.2.2 Satisfaction With the PhD Program

Satisfaction with one's PhD program was formally assessed in one study, where almost half of the respondents indicated dissatisfaction with their program in open‐ended responses (Dreifuerst et al. 2016). In this same study, 79.4% of PhD students and 86.3% of recent graduates reported starting their PhD program with the intent of becoming a nurse educator. Of those PhD graduates, 25% were currently in a full‐time faculty role (93.9% part‐time). Further, 82.2% of PhD graduates reported feeling that their program prepared them to assume a faculty role. Student satisfaction with multiple aspects of the PhD program, such as teaching preparation as discussed above, likely impacts student desire to pursue academic positions. However, a better understanding of PhD program components, and students' satisfaction with those components, is needed to determine their influence on career desires.

### 4.3 Impressions of the Faculty Role

During the PhD program, students interact with many faculty, and through these interactions, often develop impressions of nursing faculty and academia as a whole which could influence their desire to pursue an academic career. Attitudes and impressions developed towards academia may also influence students' understanding of a nurse's role or purpose within academia.

#### 4.3.1 Attitudes Towards Academia

Overall attitudes towards faculty and academia were investigated in two studies. Some who purposively sought out careers in non‐research intensive universities reported that the academic environment was not inviting (Nehls et al. 2016). How students develop these views remains an open question in the literature. Of those who self‐designated as desiring an academic career, 76.3% reported interactions with faculty as highly influential in their decision (Fang et al. 2016), suggesting positive faculty interactions or mentorship may build a positive perception of academic nursing for students. For those who did not desire an academic career, 31.7% of respondents reported that faculty interactions dissuaded them (45.5% influenced) from academic nursing. Of those in the non‐academic career group, the perceived workload of academia (43.6%) and nursing politics (54.5%) dissuaded them. While it is clear that perceptions towards academia were a factor determining career choices, it is unclear whether it is a primary factor.

#### 4.3.2 Nursing Identity/Purpose Within Academia

The perceived influential purpose of nurses within academia was a driver for some to pursue an academic career (*n* = 2 studies). In one study, a perceived impact of teaching, research, and leadership for academic nursing was associated with a 249.9% increase in the odds of planning for an academic career (Fang et al. 2016). The perceived contribution of nursing research on patient care was also highly influential. While some students report struggling with their nursing identity, specifically being in non‐clinical roles, they found support in mentors and colleagues validating the identity of nurses in academia (Xu et al. 2018). The role of the academic nurse may be confusing for nursing students at all levels and especially those in the clinical setting. Meaningful interventions may, therefore, center on de‐mystifying the role of the academic nurse, especially for undergraduate students beginning their nursing journey.

### 4.4 Program Support

Types of PhD program support, including financial support and mentorship, are likely to influence students' attitudes towards academia and their desire to pursue an academic career.

#### 4.4.1 Financial Protection

Financial protection, such as tuition coverage and stipends, was discussed in four studies. Though likely most influential in the first stage of the PhD‐Faculty pipeline when selecting a program, financial support may influence students' career desires by allowing for more attention on research. In particular, adequate financial support allows students to dedicate their time fully to coursework and research instead of continuing paid work elsewhere. In fact, one study reported that working less as an RN as a student was positively associated with having a research career after graduation (Ellenbecker et al. 2017). Only one study reported specific financial support as part of a program in Maryland which provided full tuition for all graduate programs. It is unclear how this influenced nursing PhD students, as only three students among the 250 student participants were pursuing a PhD (Daw et al. 2021). While students and graduates in another study indicated financial aid was essential for attending their PhD program, it is not clear how this influenced their career desires (Dreifuerst et al. 2016). Other programs, such as the RWJF Future of Nursing Scholars program, included funding packages (i.e., tuition and stipend) with financial matching required from individual schools (Giordano et al. 2023). Still, how this influenced career desires and eventual employment is not known.

#### 4.4.2 Multifaceted Mentorship

Mentorship, specifically faculty‐student mentorship, was discussed in multiple studies (*n* = 6) and was likely influential in other studies that did not directly discuss mentorship. One study implemented formal mentorship training for faculty who mentor PhD students as part of a large‐scale programmatic shift. More graduates in the post‐implementation group, who would have been mentored by said faculty, reported being in postdoctoral positions compared to the pre‐implementation graduates (Vance et al. 2020). In qualitative responses of students in a BSN‐entry PhD program, faculty mentorship was reported as key to becoming an independent researcher (Nehls et al. 2016) or coping with their PhD experience (Xu et al. 2018). Those who reported high levels of career readiness were more likely to have a co‐advisor compared to a single advisor (Nersesian et al. 2019). In another study, interaction with a mentor on research topics was highly influential for students who self‐reported as desiring an academic career (81.5%) and less influential for those not desiring an academic career (41.8%, neither increased or decreased desire) (Fang et al. 2016). Though not specifically discussed as it relates to career outcomes, coordinated mentorship was also part of the program curriculum of the RWJF Future of Nursing Scholars program (Giordano et al. 2023). Even considering increased involvement in research activities, it can likely be assumed that there was significant mentorship occurring to support this involvement, though this was not directly mentioned in some studies (Ellenbecker et al. 2017; Lewallen and Kohlenberg 2011). Peer mentorship, though not discussed in any of the included studies, is likely another influential factor for students' success in their program and career desires (Porat‐Dahlerbruch et al. 2021). Future research is needed in this area considering multifaceted mentorship and whether this influences future career desires.

## 5 Discussion

The purpose of this scoping review is to identify current evidence and gaps of evidence on PhD program components influencing PhD student desire to pursue academic nursing and identify existing tools, if any, to assess these relationships. This review did not identify existing standardised tools and instead reports on a myriad of tools and methods used to gather and interpret data. Through inductive content analysis, we identified multiple categories and subcategories of factors which may influence students' desire to pursue academic careers. Categories included: (1) program preparation, (2) readiness and satisfaction, (3) impressions of the faculty role, and (4) program support. Through this analysis, many gaps were identified in the existing evidence that must be addressed to more fully understand factors occurring during the PhD program that influence student career outcomes.

There were a variety of tools and methods used to gather and interpret data within the included studies. These included a mix of survey data, some with open‐ended responses, phone or video interviews, or data taken directly from schools (e.g., graduate employment data). Studies included a large variety of measures of PhD program components and career outcomes. From a synthesis standpoint, this variety renders it difficult to compare and integrate findings across multiple studies, specifically limiting our understanding of which factors that occur during the PhD program significantly influenced career outcomes. Without knowing which factors are influential, interventions cannot be developed based on evidence. Still, these findings provide a starting point to identify influential factors within PhD programs and develop a working factor inventory.

The survey tools and interview guides used in these studies included varying degrees of granularity in the questions asked. Experiences in a PhD program were often very broadly defined, such as “did you take a faculty role course?” or “did you participate in teaching, research, or development activities?,” without assessing specific activities within these experiences. Without knowing details on courses or development activities experienced by respondents, it is difficult to interpret the impact of these experiences on career outcomes or compare findings across studies. Still, some researchers assessed more specific activities such as detailed faculty role course descriptions and 3‐paper dissertations (Lewallen and Kohlenberg 2011; Vance et al. 2020). Others included study designs with participants that all participated in a similar program, such as the Maryland Support Program (Daw et al. 2021), the RWJF Future of Nursing Scholars program (Giordano et al. 2023), or being part of a BSN‐entry program (Nehls et al. 2016). Within these studies, the measure of PhD program component was simply participation in the program. Therefore, because participant experiences with specific aspects of each program were not reported, it is impossible to disentangle specific program experiences and understand which aspects of the programs were influential for career outcomes.

Overall, while multiple studies surveyed participants on whether they had a PhD program experience (i.e., did you have a faculty role course?), no studies assessed students' perceptions or experiences with these components. One study did assess whether certain experiences influenced or dissuaded participants from pursuing academia, but whether these experiences were a driving factor in decision making is unclear (Fang et al. 2016). As such, we cannot conclude whether these identified components influenced their decisions to pursue faculty roles.

Studies included a mix of prospective (e.g., current students) and retrospective (e.g., current faculty) data on career outcomes. Considering retrospective data, many studies included samples of PhD graduates asking them to think back to their PhD program experiences (Ellenbecker et al. 2017; McNelis et al. 2019). This data is limited in that retrospective data may introduce bias (Coughlin 1990), and faculty may not accurately remember all the experiences they had during their programs and how those experiences influenced their career decisions. Other studies included samples of only nursing faculty (Lee et al. 2022; Vardaman et al. 2024). These samples are further limited by only including graduates who chose to pursue academia, thus missing the potentially influential experiences of graduates who chose not to pursue academia.

Considering prospective data, one study specifically asked participants about their career plans pre‐PhD program and their change in desires after entering their PhD program (Fang et al. 2016). Another study asked participants if they began their doctoral program with the intent of becoming a nurse educator (Dreifuerst et al. 2016). In both studies, the reasons for a change in career desires were not assessed. Importantly, no study examined true longitudinal relationships, and it is unknown whether surveyed nursing PhD students eventually pursued an academic career or not. We therefore do not have the necessary data to understand how baseline career goals and attitudes change throughout the PhD program and impact later employment, and further how program components influence that change.

Empirical testing of associations between PhD program components and career outcomes was also limited. Many studies used narrative analysis or descriptive methodologies, in which percentage counts of participants who described a “program component” and then “career outcome” information were reported. Very few studies quantitatively tested associations between a PhD program component and career outcomes, and even then, the PhD program components often lacked specificity, limiting interpretation. While narrative and descriptive data clarify potential influential factors, without modelling associations, it is difficult to understand true associations between these factors and career outcomes.

The categories and influential factors identified in this scoping review are limited by the existing literature, which, as highlighted above, includes notable gaps. It is likely there are other influential factors not captured in our results. Some notable areas or influential factors may include constellation mentorship, which includes peer and faculty mentorship (Porat‐Dahlerbruch et al. 2021), leading a team on an independent research project (Killela et al. 2024), mentorship and leadership training (Abbott‐Anderson et al. 2016; Han et al. 2022), experiences of minority students (Avery‐Desmarais et al. 2021), and job and family responsibilities (Williams et al. 2021). Further research is needed to identify true influential factors on students' career desires, with a particular emphasis on current students' perceptions of their experiences in the PhD program and how/if these influence career desires. From this research, a factory inventory can be developed, which includes PhD program factors that are influential for students' career desires and eventual employment in academia. This scoping review is an initial step in developing this factor inventory.

### 5.1 Future Directions

In the future, a comprehensive list of factors influencing students' desire to pursue academic careers can be used to develop a standardised survey tool which assesses the second stage of the PhD‐Faculty pipeline, including specific program components, student perceptions of those components, and their influence on self‐reported career goals/attitudes and actual employment. This tool should assess students' career goals upon entering and throughout the PhD program, capture measurements of both program experiences and students' perception of those experiences, and follow students as they graduate and become employed. This will allow for more effective tracking of actual transition from PhD student to a potential faculty role.

Such a survey tool is a critical step in (1) improving our understanding of which program components may influence or dissuade PhD students from pursuing academic careers and (2) developing interventions which target the second stage of the PhD‐Faculty pipeline. With a standardised survey tool, the gaps highlighted in this scoping review can be addressed, and the associations between program components and career outcomes can be tested and interpreted. This survey tool could be used by individual schools or multi‐institutional studies, allowing for broad synthesis of results across institutions and studies. Findings could in turn help inform recommendations for schools which may increase the number of PhD nursing students who desire and eventually pursue academic careers.

### 5.2 Limitations

First, our search may have excluded studies which included data relevant to the research question but were not captured by the search terms. Still, as this was a scoping review, our goal was to clarify influential factors and identify gaps that can inform future investigation (Munn et al. 2018). Second, some studies were excluded that described a PhD program component but did not include a measure of career outcomes. For example, one study detailed the implementation of a research immersion project but did not report outcomes related to career desires, attitudes, or employment (Killela et al. 2024). Program components described in excluded studies may be influential for career outcomes and should be considered in future work developing an influential factor inventory. Third, there are many other experiences that PhD students have outside the PhD program itself, including personal lives and family, individual finances, and geographic restraints. Our review did not aim to capture these aspects of the PhD experience. Nevertheless, we recommend that future research integrates a standardised survey tool to assess program‐related experiences, which can capture more personal experiences during the PhD period and harmonise that data to inform future recommendations. Fourth, while we did not exclude studies based on geographic location, the only studies that met our inclusion criteria were conducted in the United States. One such excluded study was a multi‐country assessment of faculty, student, and graduate perceived quality of their PhD program (Kim et al. 2015). While this study provides useful insights into the impact of certain factors of the program (e.g., resources) on perceptions of quality, links with career goals and employment were not made. Future research should be conducted in multiple countries to improve generalizability of these influential factors. Finally, while this review focuses on the importance of strengthening the PhD‐Faculty pipeline, we recognise there are additional critical roles PhD prepared nurses can pursue beyond a faculty role (McKenna and Thompson 2025; Polomano et al. 2021).

## 6 Conclusion

In conjunction with declining PhD enrollment and graduation rates (Halabicky et al. 2024), fewer PhD prepared nurses are choosing to pursue academic faculty roles. It is likely that factors occurring in the second stage of the PhD‐Faculty pipeline, during the PhD program, influence students' desire and eventual decision to pursue academia. Identifying these influential factors will improve our understanding of what components of the PhD program influence current students and provide evidence which informs interventions to target the second stage of the PhD‐Faculty pipeline. This scoping review identified four categories of influential factors including (1) program preparation, (2) readiness and satisfaction, (3) impressions of the faculty role, and (4) program support. Still, there were many gaps in the existing literature including a large variety of study designs and methods, lack of specificity in program experiences, and few studies which tested formal associations between PhD program components and career outcomes.

There is much work to be done to fully understand how experiences during the PhD program influence a PhD student's career goals and later employment as nursing faculty. This scoping review provides a foundation for a program of study identifying influential factors within PhD programs which influence PhD students' career desires. By developing a robust factor inventory and eventually a standardised survey tool, individual schools and multi‐institution studies can collect data on program components, student's perceptions of and experience with those components, and their career outcomes (i.e., goals, attitudes, employment) over time, as well as assessment of associations between these concepts. Knowledge gained through such a tool will help to develop and test programmatic interventions to improve the second stage of the PhD‐Faculty pipeline, thus addressing the nursing faculty shortage.

## Funding

The authors have nothing to report.

## Ethics Statement

This study did not require ethical approval by an Institutional Review Board and does not include human subjects. This study adheres to PRISMA extension guidelines for scoping reviews.

## Conflicts of Interest

The authors declare no conflicts of interest.

## Supporting information


**Data S1:** PRISMA Checklist.

**Table S1:** Search Terms and Search Strategy.

## Data Availability

Data sharing not applicable to this article as no datasets were generated or analysed during the current study.
