# Supplementary material for: What We Know—and Need to Know—About Nursing PhD Programs and Influences on the PhD–Faculty Pipeline: A Scoping Review
Source: J Adv Nurs. 2026 Jan 30;82(5):4134–52. doi: 10.1111/jan.70506 (PMC13069202; doi:10.1111/jan.70506)
Supplement: Supplementary file 2 — Table S1: Search Terms and Search Strategy. [file JAN-82-4134-s001.docx]

**Supplemental Table 1** Search Terms and Search Strategy

| **Concept** | **Date** | **Search** |
| --- | --- | --- |
| PubMed | 10.3.2025 | ("PhD education"[All Fields] OR "nurs* phd"[All Fields] OR "PhD in nursing"[All Fields] OR "phd prepared nurse"[All Fields]) AND ("faculty, nursing"[MeSH Major Topic] OR "Faculty"[MeSH Terms] OR "nursing faculty"[All Fields] OR "university faculty"[All Fields] OR "assistant professor"[All Fields] OR "professor"[All Fields]) |
| CINAHL | 10.3.2025 | "PhD education" OR "nurs* phd" OR "PhD in nursing" OR "phd prepared nurse"  AND  (MH "Faculty") OR (MH "Faculty, Nursing")  OR "nursing faculty" OR "university faculty" OR "assistant professor" OR "professor" |
| Scopus | 10.3.2025 | ( TITLE-ABS-KEY ( "nurs* faculty" ) OR "assistant professor" ) AND ( "PhD education" OR "nurs* phd" OR "PhD in nursing" OR "phd prepared nurse" ) |
